# Advanced diffuse hepatic angiosarcoma treated successfully with TACE and targeted immunotherapy: A case report

**DOI:** 10.3389/fonc.2023.1071403

**Published:** 2023-04-20

**Authors:** Yucheng Lin, Zheng Chen, Jianchuan Yang, Ying Lin, Sheng Chen, Ying Xie, Songsong Wu

**Affiliations:** ^1^ Department of Ultrasonography, Fuzhou No. 1 Hospital Affiliated with Fujian Medical University, Shengli Clinical Medical College, Fuzhou, China; ^2^ Department of Oncology, Fujian Provincial Hospital, Shengli Clinical Medical College of Fujian Medical University, Fuzhou, China; ^3^ Department of Ultrasonography, Fujian Provincial Hospital, Shengli Clinical Medical College of Fujian Medical University, Fuzhou, China; ^4^ Department of Pathology, Fujian Provincial Hospital, Shengli Clinical Medical College of Fujian Medical University, Fuzhou, China; ^5^ Department of Hematology, Fujian Provincial Hospital, Shengli Clinical Medical College of Fujian Medical University, Fuzhou, China

**Keywords:** hepatic angiosarcoma, interventional treatment (TACE), immunotargeted therapy, camrelizumab, anlotinib

## Abstract

Primary hepatic angiosarcoma (PHA), a rare soft tissue tumor, accounts for only 2% of all liver malignancies. Pathologically challenging, PHA is difficult to be distinguished from other malignancies with ultrasound, Computed Tomography (CT), or Magnetic Resonance Imaging (MRI). Due to late diagnosis and resistance against traditional chemotherapy and/or radiotherapy, only 3% of PHA patients can survive up to two years after diagnosis. To our best knowledge, this case report presents the first case of an advanced diffuse PHA with ruptured hemorrhage that has been effectively treated with TACE and Anlotinib plus Camrelizumab. So far, the patient has received 10 cycles of treatment and is faring well. Latest MRI results show that the tumor has shrunk by 56% and can be assessed as a partial response (PR). This case report includes our experience in treating such a advanced malignancy, and we hope that larger studies on advanced PHA can better quantify the potential benefit.

## Introduction

Primary hepatic angiosarcoma (PHA) is a rare soft tissue tumor (1) that grows rapidly and features frequent rupture and hemorrhage (2). PHA is resistant against traditional chemotherapy and/or radiotherapy, while its clinical symptoms and radiographic examination results are generally non-specific, making early diagnosis more difficult (3–5). In most cases, patients cannot be diagnosed soon enough for surgery, which is considered the most effective treatment (6, 7). Due to the small number of PHA cases and rapid disease progression, only a few studies in this field have been published and treatment is not yet standardized. Therefore, improved treatment strategies are urgently needed. Transcatheter arterial chemoembolization (TACE) has been proved to effective in emergency control of tumor rupture and bleeding (8). Immunotherapy targeting programmed death 1 (PD-1) receptor and its ligand (PD-L1) has recently been found to have activity in multiple cancers (9). Anlotinib, a novel oral multi-targeted receptor tyrosine kinase inhibitor (TKI), has a broad spectrum of inhibitory action on tumor angiogenesis and growth (10), and showed positive effects (11) as a monotherapy and in a combination therapy for advanced sarcoma. To our best knowledge, this case report presents the first case of an advanced diffuse PHA with ruptured hemorrhage that has been effectively treated with TACE and Anlotinib plus Camrelizumab, as well as includes our detailed experience in treating such a rare malignancy.

## Case presentation

**Figure 1:**
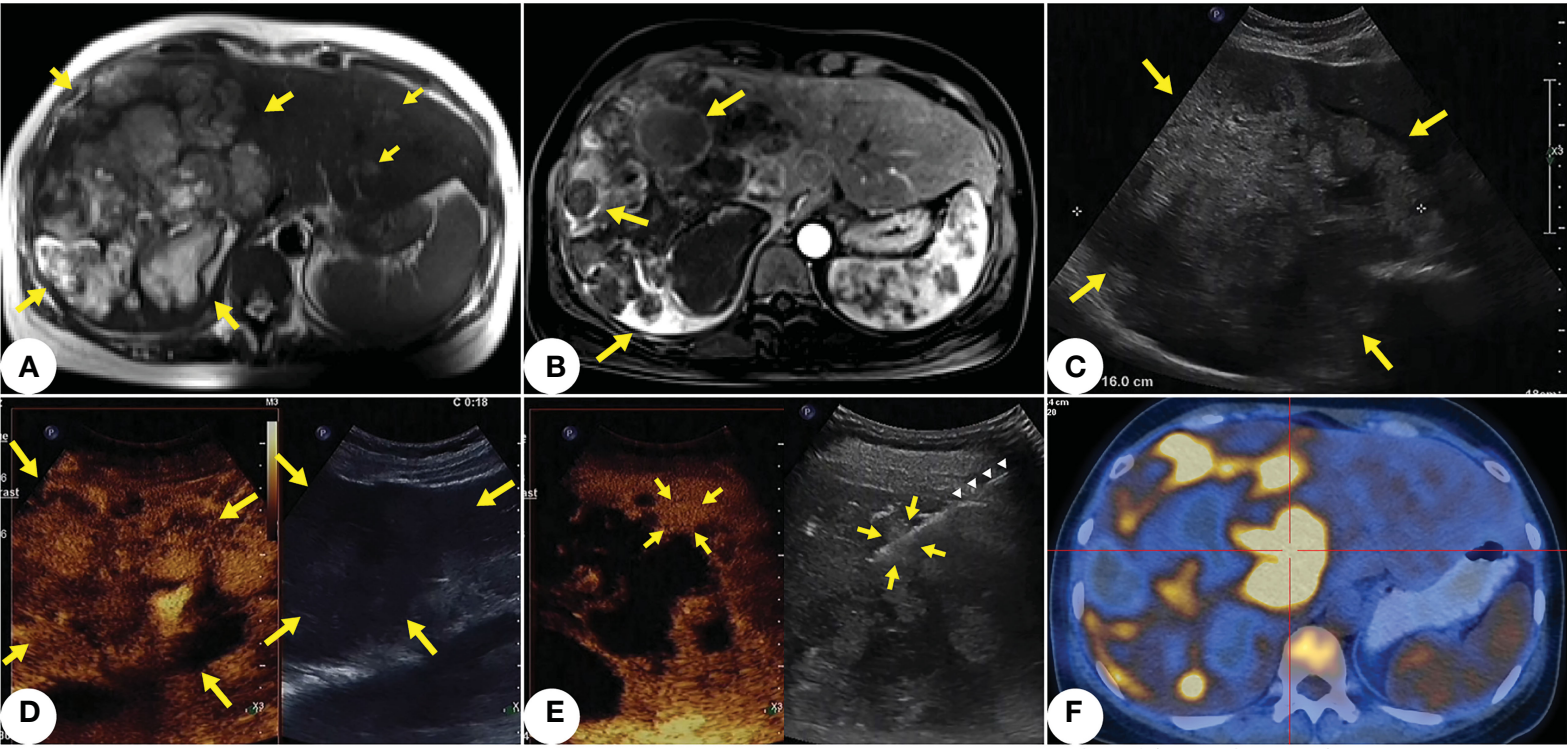
Primary hepatic angiosarcoma (PHA) in a 42-years-old woman. **(A)** T2-weighted axial MRI shows multiple variable-sized lesions with bright T2 signal intensity are located in both hepatic lobes. Mass lesions of PHA reveal a markedly heterogeneous architecture, with focal areas of high intensity along with septum-like or rounded areas of low intensity (indicated by yellow arrows). **(B)** Some lesions show annular enhancement at the edges; large masses show ruptures and hemorrhages (indicated by yellow arrows). **(C)** Ultrasound shows a 16cm inhomogeneous hypoechoic lesion in the right hepatic lobe (indicated by yellow arrows). Multiple masses scatter within both hepatic lobes. **(D)** Contrast-enhanced ultrasound shows the lesion (indicated by yellow arrows) in ill-defined, peripheral irregular isoenhancement, and necrotic and hemorrhagic areas within the tumor. **(E)** After selecting a target lesion with multimodal enhanced imaging, (indicated by yellow arrows), we performed core needle biopsy (indicated by white triangles) under the guidance of contrast-enhanced ultrasound. **(F)** PET-CT shows multiple hypermetabolism lesions with retroperitoneal lymph node metastasis in the liver.

A 42-year-old woman with anemia and fatigue resorted to our hospital in March 2021. She had mild bloating but no significant weight loss. Examination showed that her abdomen was soft with no upper quadrant tenderness, and there was no sign of peritonism. Initial laboratory results showed her hemoglobin (Hb) at 65 g/L, while liver function tests showed her alanine transaminase (ALT) at 28 units/L and her aspartate transaminase (AST) at 46 units/L. Nothing alarming was found in tumor markers, infection serology, or the patient’s medical records and family history. On day 1 of hospitalization, initial MRI of abdomen revealed that the patient had a large (16cm), irregular, dominant mass with multiple intrahepatic lesions in her liver, as well as trace free fluid in her abdominal cavity (**Figures 1A, B**). Gray-scale ultrasound and contrast-enhanced ultrasound showed multiple masses scattered in both hepatic lobes (**Figures 1C, D**), and there were necrotic and hemorrhagic areas within the tumor. Our clinician requested biopsy to clarify the pathology of the lesion. After assessing the situation with multimodal enhanced imaging, we performed percutaneous core needle biopsy for a small lesion that was active and surrounded by liver parenchyma under the guidance of contrast-enhanced ultrasound (**Figure 1E**). After the biopsy, thrombin agent was injected into the needle track to reduce or even stop possible bleeding. On day 2, positron emission tomography (PET-CT) for staging showed multiple hypermetabolism lesions with retroperitoneal lymph node metastasis in the patient’s liver (**Figure 1F**).

**Figure 2:**
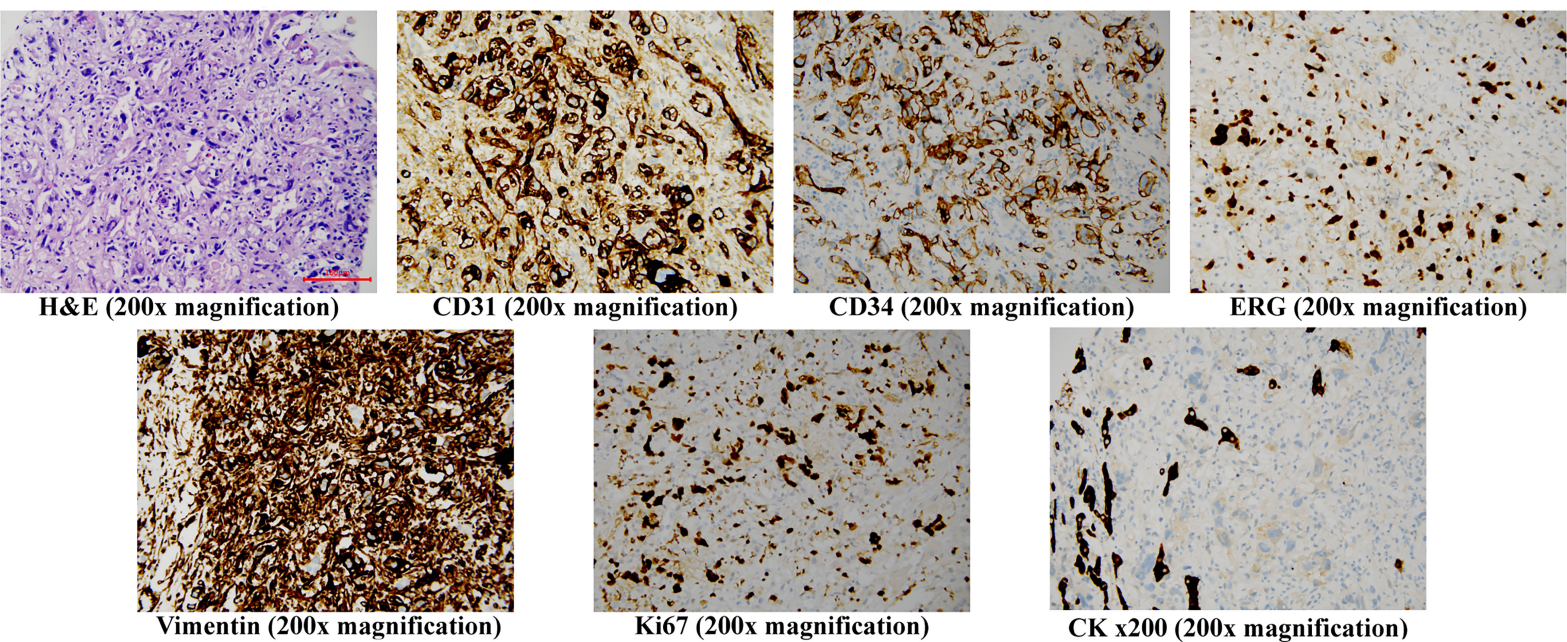
Histopathology staining of the core needle liver biopsy specimen H&E (200x magnification) shows cells with pleomorphic nuclei and frequent mitotic figures, solid nodules of similar atypical spindled endothelial cells and focal areas of with epithelioid morphology. Immunohistochemistry (200x magnification) shows the cells to be positive for CD31, CD34, ERG, Vimentin and Ki67, but negative for CK, H&E, hematoxylin and eosin.

On the late night of day 3, the patient experienced sudden upper abdominal pain and fever (maximum body temperature: 39°C). Emergency ultrasound showed tumor rupture with light haemoperitoneum, but her blood count and liver function did not change significantly from those upon hospitalization. Therefore, we opted for physical cooling and carried out protection of liver function with polyenyl phosphorylcholine 500mg slow intravenous drop infusion plus 1200mg glutathione intravenous drip every day (QD). And we prepared the patient for multidisciplinary treatment. On day 4, histological examination showed cells with pleomorphic nuclei and frequent mitotic figures, solid nodules of similar atypical spindled endothelial cells and focal areas with epithelioid morphology. Immunohistochemical examination revealed positive staining of CD31(+), CD34(+), ERG(+), CK (–), Vimentin(+), Ki67(70%+), indicating tumor cells (**Figure 2**). The histopathological diagnosis confirmed PHA.

**Figure 3:**
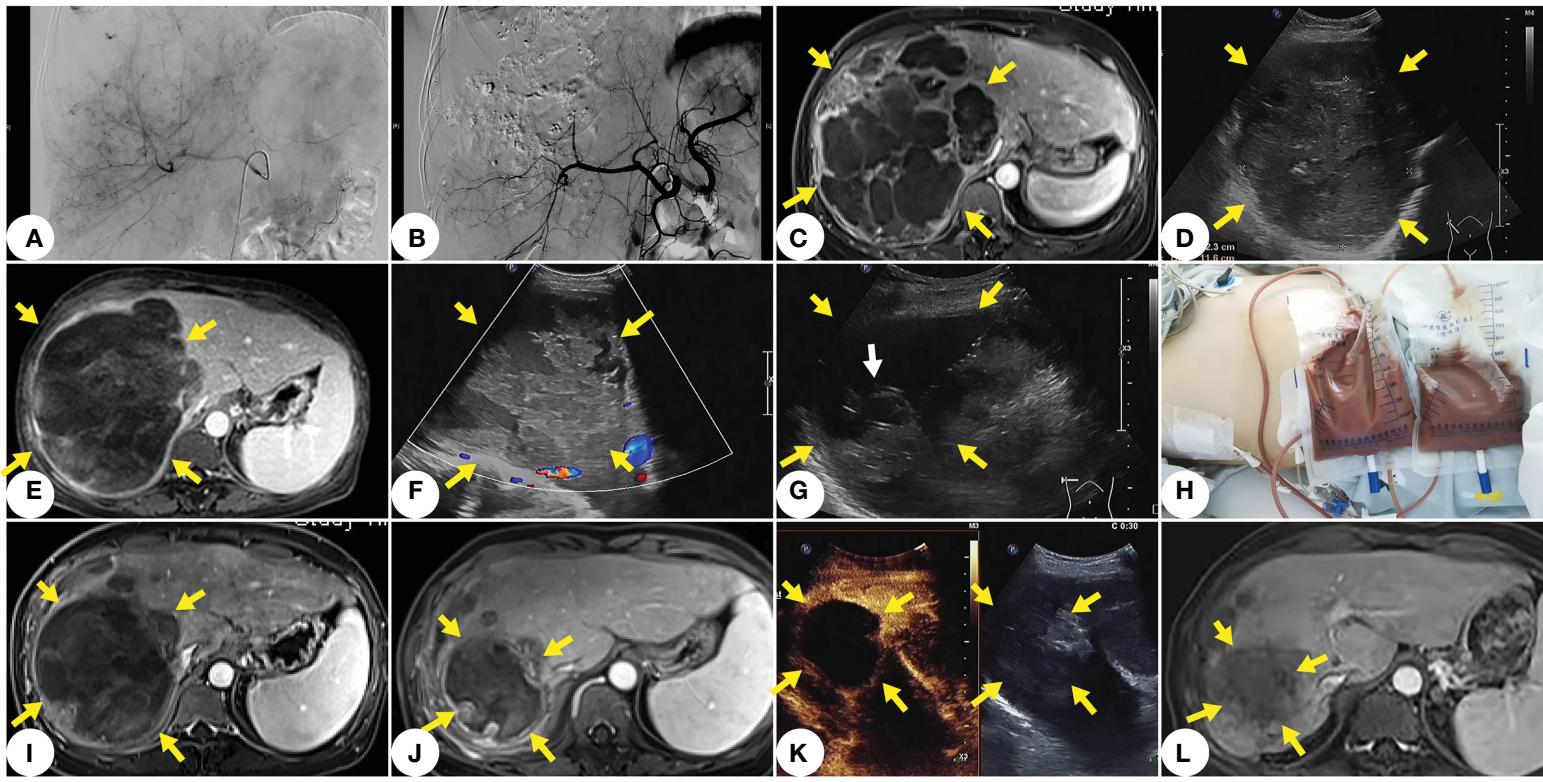
The main treatment and imaging of this case. **(A)** Tumor imaging stains visible under DSA before TACE. **(B)** No tumor staining under DSA after TACE. **(C)** MRI one month after TACE shows no significant tumor progression. **(D)** Ultrasound two months after TACE shows slightly shrunk tumor. **(E)** MRI after the 8th cycle of treatment shows that the onset of tumor liquefaction, and necrosis and disintegration. **(F)** Ultrasound clearly shows tumor liquefaction and necrosis. **(G)** Ultra- sound-guided percutaneous catheter punctures into the pus and necrosis at the internal drainage site of the tumor (white arrow indicates the end of the drainage catheter). **(H)** A large amount of brown serous pus fluid drained with percutaneous catheter from the liquefied area of the thorax and tumor. **(I)** MRI after thoracic and abdominal drainage. **(J)** MRI at 15 months after diagnosis of PHA. **(K)** Contrast-enhanced ultrasound image at 15 months after diagnosis of PHA shows significant shrinkage of the main lesion without enhancement and no new lesions in the liver. **(L)** MRI at two years after diagnosis of PHA shows that shrunk lesions, and no activity or new lesion.

Subsequently, we conducted a multidisciplinary joint consultation to devise the treatment plan of low-dose TACE for controlling tumor bleeding and tumor progression. After assessing the patient’s disease status as stable, we carried out treatment with 200mg Camrelizumab every three weeks plus 8mg Anlotinib QD. Chemoembolization was performed with an oil-based mixture (doxorubicin mixed with ethiodized oil), followed by infusion of Embospheres (300μm-500μm particles). Up to 30mg of doxorubicin was used in chemoembolization. After the first TACE treatment, the patient’s liver function declined transiently. She had recurrent fever with a body temperature of up to 39°C, and CT results showed pulmonary infection. The patient’s fever was under well control after administering Tienam for three weeks, and her liver function abnormalities disappeared. Then, we started the regimen of Camrelizumab plus Anlotinib, which was well tolerated by the patient. After the second low-dose TACE treatment at a 4-week interval, the patient did not suffer fever or declined liver function. So far, the patient has received 10 cycles of treatment and is faring well. The only side effect was hypothyroidism, which was easily controlled by our multidisciplinary treatment team. The patient’s PET-CT results after the 4th cycle of treatment showed tumor shrinkage, which was then assessed as a stable disease (SD) by our oncologist after the 6th cycle of treatment. Contrast-enhanced ultrasound results after the 8th cycle of treatment indicated significant tumor reduction (8cmx9cm). During this period, however, the patient again suffered sudden abdominal pain and fever. Ultrasound results showed liquefaction, necrosis and local rupture of the lesion, and a diagnostic puncture suggested liver abscess, indicating the onset of tumor disintegration. We drained a large amount of brown serous pus fluid and subsequent abdominal and pleural effusions from the patient with percutaneous catheter, and adopted anti-infective and liver-protective treatments (polyenyl phosphorylcholine, 500mg, QD, slow intravenous drop infusion, plus glutathione, 1200 mg, QD, intravenous drip). The patient’s repeated pulmonary infection during this period was effectively treated with high-grade antibiotics. The entire drainage process continued intermittently for one month; the fluid drained from the patient’s thoracic cavity, abdominal cavity and liver abscess totaled 14,800ml. After 17 months of treatment, the patient’s follow-up MRI results showed that the tumor had shrunk to 7cm with internal coagulation necrosis, no active areas in the lesions, and no liver abscess (**Figure 3**).

**Figure 4:**
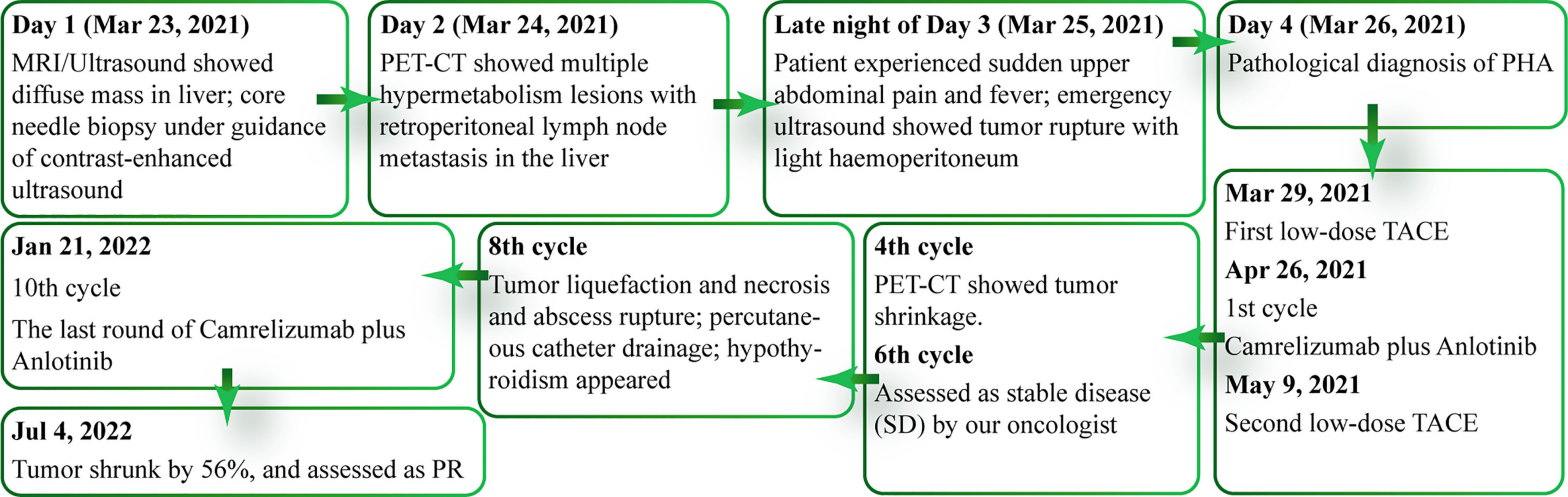
Timeline of patient's clinical course.

After 17 months of treatment, the patient’s tumor has shrunk by 56% and been assessed as a PR. From making a definite diagnosis to now, two years have passed, she now enjoys a high-quality life without further tumor progression or other related ailments, and has even gained some weight. The patient’s clinical course timeline is shown in **Figure 4**.

## Discussion

PHA is a rare malignant vascular tumor that is aggressive and often based on malignant endothelial origin involving blood and lymph vessels (1, 12). PHA does not have representative clinical features, and has the common symptoms (e.g., abdominal pain, weakness and weight loss) of most malignant wasting diseases; PHA patients are often tested negative for tumor markers (AFP, CEA, CA19-9 and CA125), and usually have no history of toxic exposure (7).Accurate imaging diagnosis and tumor staging bring good prognosis (13), especially for patients with advanced PHA. Regrettably, most PHA cases detected with initial imaging are multiple nodules of varied sizes, large solitary masses, or mixed masses of predominant masses and nodules. Due to overlapping imaging features (14), it is even difficult for enhancement imaging to distinguish the malignant features of PHA from those of other malignancies (e.g., epithelioid hemangioendothelioma and hepatic cholangiocarcinoma). Even when it was accidentally discovered, PHA can be misdiagnosed as hemangioma at an early stage until it is surgically removed to stop excessive rapid growth (15).

Since pathologic diagnosis is golden standard for identifying PHA, and a rapid & accurate pathological diagnosis is required to address the concerns about tumor progression, we recommended an efficient approach—percutaneous core needle liver biopsy, which can provide pathological evidence for guiding treatment strategy (16). After assessing the situation with multimodal enhanced imaging, we performed percutaneous core needle biopsy for a small lesion that was active and surrounded by liver parenchyma under the guidance of contrast-enhanced ultrasound, avoiding the dominant necrotic mass and the vulnerable area of the tumor. After the biopsy, thrombin agent was injected into the needle track to reduce or even stop possible bleeding (17, 18). Histologically, hepatic angiosarcoma manifests as cells with pleomorphic nuclei and frequent mitotic figures, solid nodules of similar atypical spindled endothelial cells and focal areas of with epithelioid morphology. Histopathology shows a variety of patterns of vascular channels, dilated sinusoidal or cavernous spaces. The dilated and congested sinusoidal spaces were lined by atypical cells with enlarged hyperchromatic nuclei. This structure could have resulted in were generated and a large amount of platelets and coagulation factors were consumed in the tumor, leading to disseminated intravascular coagulopathy (DIC) (19). Tumors are immunoreactive to CD31, CD34, ERG, Ki-67, and pancytokeratin (CK). VEGFR2 is strong in most angiosarcomas, representing the basis for using targeted therapy with VEGFR2 inhibitors in the treatment of angiosarcomas according to reports (20, 21). Even if pathologically diagnosed, some advanced cases progress so fast that patients don’t even have the chance to receive any treatment. For our patient, we completed all examinations, including a percutaneous biopsy under the guidance of contrast-enhanced ultrasound, within 72 hours after she was hospitalized. Such prompt efforts will contribute to early diagnosis of advanced diseases for high-grade tumor burden patients (1, 22).

Due to the tumor biology of PHA, the tumor relapses very quickly after surgery (23, 24). TACE is often used urgently for treating the rupture and bleeding of tumors in PHA patients, while local chemotherapy can effectively prolong the life of patients and potentially control tumor growth and metastasis. These measures can prevent fatal tumor rupture and massive hemorrhage, especially in patients with a large tumor burden (25). Due to vigilance about tumor aggravation after TACE (8), we used a low-dose drug (30mg of doxorubicin, half of conventional dose) for TACE to prevent liver failure, and actively treated the patient with liver-protective drugs after embolization (polyenyl phosphorylcholine 1000mg slow intravenous drop infusion plus 2400 mg glutathione intravenous drip QD). TACE inhibits the further rapid progress of the lesions in the short term, creating the opportunity for targeted immunotherapy to extend the patient’s survival time (26). After the tumor dissolved, we used ultrasound-guided percutaneous catheter to drain the necrotic material and pus in a timely manner. Such a procedure was important for the patient’s prognosis, since it helped to remove toxic necrotic fluid and reduce the tumor size/burden, that improves the effectiveness of the overall treatment plan. Meanwhile, we actively dealt with complications (decline of liver function, pulmonary infection, abdominal and pleural effusions, etc.). Multidisciplinary treatment has a positive effect on the prognosis of tumors.

Systemic therapy appears to be the only option for patients with advanced PHA. Chemotherapeutic agents for treatment of sarcomas did not improve the patient’s survival time until the development of targeted therapy and immunotherapy in recent years (27, 33). Therefore, we did not try chemotherapy due to concerns about the patient’s inability to tolerate it. Camrelizumab, a newly developed humanized PD-1 monoclonal antibody, binds to PD-1 with high-affinity to block the PD-1 pathway on T cells (28). the synergistic effect of Anlotinib plus Camrelizumab However, the number of PHA patients is too small to make definite conclusions association between anti-tumour activity and higher PD-L1 expression on tumor cells (29, 30). The report of Liu et al (29) also showed Camrelizumab has shown encouraging activity in combination with anlotinib in the treatment of multiple tumorsi including soft tissue sarcoma(STS). Anlotinib, a novel oral multi-targeted receptor TKI, acts on a proven target’s vascular endothelial growth factor (VEGF) isoforms and their receptors (VEGFRs), thereby demonstrating significant antitumor effects through the inhibition of angiogenetic and proliferative signaling. Anlotinib is approximately 20-500 times as potent as comparable TKIs (10, 11). Yao et al (11) reported in 2022 that Anlotinib as a monotherapy or in combination therapy can be more effective and safer for certain advanced sarcomas. Usually a reduction in the diameter of the target lesion about 1 month after the therapy, but certain disappeared lesions reappear and progress rapidly, a phenomenon that might be related to drug resistance or insufficient antitumor effect. Hypertension, hand-foot syndrome, weakness, myelosuppression and hypothyroidism can appear after immunotherapy is combined when using Anlotinib. The main problems with both drugs are short duration with secondary drug resistance. TKIs block these proangiogenic pathways, promote vessel normalization, improve tumor perfusion and oxygenation, restore the hypoxic tumor microenvironment, and enhance drug delivery. Meanwhile, the combined antiangiogenic and anti-PD-1/PD-L1 therapy has been shown to elicit T- cell function and drive tumor cells to activate immune checkpoints, resulting in greater antitumor immunity than anti-PD-1 treatment alone (27, 31, 32). Therefore, we used the combination program of Camrelizumab plus Anlotinib to try to delay the patient’s condition. Patients tolerated it well, and this may be an effective treatment option, but further large-scale comparative studies are required. Zhou et al (33) reported some encouraging activity with Anlotinib combined with anti-PD-1 antibody, Camrelizumab, for advanced non-small-cell lung cancers (NSCLCs) in 2021.

So far, only a small number of patients have received TACE to control tumor rupture and bleeding before targeted therapy. However, they were treated with conventional doses of TACE, and the combination of Anlotinib and PD1 has been rare. Their tumor burdens were much smaller, and their cases cannot be followed up today (22, 29, 31). Our case, typical and successful, provides positive evidence for the combination of both agents in patients with advanced PHA. Anlotinib plus Camrelizumab, well tolerated and with acceptable complications, has demonstrated encouraging efficacy in treating advanced PHA.

## Conclusion

The combination of TACE and targeted immunotherapy may be an effective way to treat advanced diffuse hepatic angiosarcoma. Early assessment with multimodal imaging and percutaneous core needle biopsy for pathological diagnosis are conducive to timely treatment, while promptly multidisciplinary treatment of complications during tumor necrosis can help improve prognosis. Our case report aims to encourage further studies on treatment of advanced PHA.

## Data availability statement

The original contributions presented in the study are included in the article/supplementary material. Further inquiries can be directed to the corresponding authors.

## Ethics statement

Written informed consent was obtained from the participant/patient(s) for the publication of this case report.

## Author contributions

YuL, ZC, JY, YiL have contributed equally to this work and share first authorship. WS and XY are co-corresponding authors. All authors contributed to the article and approved the submitted version.

## References

[1] Mani H Van Thiel DH. Mesenchymal tumors of the liver. Clin Liver Dis (2001) 5(1):219–57, viii. doi: 10.1016/s1089-3261(05)70162-8 11218917

[2] Matthaei H Krieg A Schmelzle M Boelke E Poremba C Rogiers X. Long-term survival after surgery for primary hepatic sarcoma in adults. Arch Surg (2009) 144(4):339–44. doi: 10.1001/archsurg.2009.30 19380647

[3] Cazorla A Félix S Delabrousse E Valmary-Degano S. [Primary hepatic angiosarcoma: a retrospective analysis of eight cases]. Ann Pathol (2014) 34(6):462–8. doi: 10.1016/j.annpat.2014.10.004 25499861

[4] Yi L-L Zhang J-X Zhou S-G Wang J Huang Y-Q Li J. CT and MRI studies of hepatic angiosarcoma. Clin Radiol (2019) 74(5):406.e1–8. doi: 10.1016/j.crad.2018.12.013 30686504

[5] Wang L Lv K Chang XY Xia Y Yang ZY Jiang YX. Contrast-enhanced ultrasound study of primary hepatic angiosarcoma: a pitfall of non-enhancement. Eur J Radiol (2012) 81(9):2054–9. doi: 10.1016/j.ejrad.2011.06.026 21737220

[6] Chaudhary P Bhadana U Singh RAK Ahuja A. Primary hepatic angiosarcoma. Eur J Surg Oncol (2015) 41(9):1137–43. doi: 10.1016/j.ejso.2015.04.022 26008857

[7] Kim HR Rha SY Cheon SH Roh JK Park YN Yoo NC. Clinical features and treatment outcomes of advanced stage primary hepatic angiosarcoma. Ann Oncol (2009) 20(4):780–7. doi: 10.1093/annonc/mdn702 19179547

[8] Park YS Kim JH Kim KW Lee IS Yoon H-K Ko G-Y. Primary hepatic angiosarcoma: imaging findings and palliative treatment with transcatheter arterial chemoembolization or embolization. Clin Radiol (2009) 64(8):779–85. doi: 10.1016/j.crad.2009.02.019 19589416

[9] Sindhu S Gimber LH Cranmer L McBride A Kraft AS. Angiosarcoma treated successfully with anti-PD-1 therapy - a case report. J Immunother Cancer (2017) 5(1):58. doi: 10.1186/s40425-017-0263-0 28716069 PMC5514460

[10] Sun Y Niu W Du F Du C Li S Wang J. Safety, pharmacokinetics, and antitumor properties of anlotinib, an oral multi-target tyrosine kinase inhibitor, in patients with advanced refractory solid tumors. J Hematol Oncol (2016) 9(1):105. doi: 10.1186/s13045-016-0332-8 27716285 PMC5051080

[11] Yao W Du X Wang J Wang X Zhang P Niu X. Long-term efficacy and safety of anlotinib as a monotherapy and combined therapy for advanced sarcoma. Onco Targets Ther (2022) 15(undefined):669–79. doi: 10.2147/OTT.S365506 PMC9206457 35726279

[12] Wilson GC Lluis N Nalesnik MA Nassar A Serrano T Ramos E. Hepatic angiosarcoma: a multi-institutional, international experience with 44 cases. Ann Surg Oncol (2019) 26(2):576–82. doi: 10.1245/s10434-018-7062-9 30456677

[13] Tsunematsu S Muto S Oi H Naka T Kitagataya T Sasaki R. Surgically diagnosed primary hepatic angiosarcoma. Intern Med (2018) 57(5):687–91. doi: 10.2169/internalmedicine.9318-17 PMC5874340 29151516

[14] Alexander LF Harri P Little B Moreno CC Mittal PK. Magnetic resonance imaging of primary hepatic malignancies in patients with and without chronic liver disease: a pictorial review. Cureus (2017) 9(8):e1539. doi: 10.7759/cureus.1539 28989828 PMC5628780

[15] Yuan WH Li AF Hsu HC Hu YS Lee RC. Initial clinical radiological findings and staging to predict prognosis of primary hepatic angiosarcoma: a retrospective analysis. PloS One (2019) 14(11):e0225043. doi: 10.1371/journal.pone.0225043 31710641 PMC6844487

[16] Almogy G Lieberman S Gips M Pappo O Edden Y Jurim O. Clinical outcomes of surgical resections for primary liver sarcoma in adults: results from a single centre. Eur J Surg Oncol (2004) 30(4):421–7. doi: 10.1016/j.ejso.2004.01.004 15063896

[17] Drinković I Brkljacić B. Two cases of lethal complications following ultrasound-guided percutaneous fine-needle biopsy of the liver. Cardiovasc Intervent Radiol (1996) 19(5):360–3. doi: 10.1007/BF02570192 8781161

[18] Kang TW Lee MW Choi D An C Kim MJ Joo I. Safety of percutaneous biopsy for hepatic angiosarcoma: results of a multicenter Korean survey. J Vasc Interv Radiol (2016) 27(6):846–51. doi: 10.1016/j.jvir.2016.01.148 27080009

[19] Fujii F Kimura T Tanaka N Kubota D Sugiura A Umemura T. Hepatic angiosarcoma with kasabach-Merritt phenomenon: a case report and review of the literature. Ann Hepatol (2018) 17(4):655–60. doi: 10.5604/01.3001.0012.0949 29893706

[20] Yasir S Torbenson MS. Angiosarcoma of the liver: clinicopathologic features and morphologic patterns. Am J Surg Pathol (2019) 43(5):581–90. doi: 10.1097/PAS.0000000000001228 30986799

[21] Wang Z-B Yuan J Chen W Wei L-X. Transcription factor ERG is a specific and sensitive diagnostic marker for hepatic angiosarcoma. World J Gastroenterol (2014) 20(13):3672–9. doi: 10.3748/wjg.v20.i13.3672 PMC3974537 24707153

[22] Pierce DB Johnson GE Monroe E Loggers ET Jones RL Pollack SM. Safety and efficacy outcomes of embolization in hepatic sarcomas. AJR Am J Roentgenol (2018) 210(1):175–82. doi: 10.2214/AJR.16.17573 29090997

[23] Lin Y-H Lin C-C Concejero AM Yong C-C Kuo F-Y Wang C-C. Surgical experience of adult primary hepatic sarcomas. World J Surg Oncol (2015) 13(undefined):87. doi: 10.1186/s12957-015-0489-6 25880743 PMC4358880

[24] Kim B Reardon R Cross J Fehlberg T Allanson B Punch GJ. Case report: haemoperitoneum secondary to acute rupture of primary hepatic angiosarcoma. Int J Surg Case Rep (2021) 84(undefined):106090. doi: 10.1016/j.ijscr.2021.106090 34139418 PMC8219754

[25] Jiang S Wu H Lu M Li N. Surgery and chemotherapy improve the prognosis of primary hepatic angiosarcoma: a retrospective study based on propensity score matched survival analysis. Eur J Surg Oncol (2021) 47(null):690–8. doi: 10.1016/j.ejso.2020.11.121 33239254

[26] Testa S Bui NQ Wang DS Louie JD Sze DY Ganjoo KN. Efficacy and safety of trans-arterial yttrium-90 radioembolization in patients with unresectable liver-dominant metastatic or primary hepatic soft tissue sarcomas. Cancers (Basel) (2022) 14(2):324. doi: 10.3390/cancers14020324 35053486 PMC8774147

[27] Yi M Zheng X Niu M Zhu S Ge H Wu K. Combination strategies with PD-1/PD-L1 blockade: current advances and future directions. Mol Cancer (2022) 21(1):28. doi: 10.1186/s12943-021-01489-2 35062949 PMC8780712

[28] Xu Z Zhang Y Yu Y-H. Successful treatment of advanced alveolar soft part sarcoma with camrelizumab combined with apatinib: a case report. Ann Palliat Med (2021) 10(1):785–92. doi: 10.21037/apm-20-2275 33545800

[29] Liu J Gao T Tan Z Li S Xu J Bai C. Phase II study of TQB2450, a novel PD-L1 antibody, in combination with anlotinib in patients with locally advanced or metastatic soft tissue sarcoma. Clin Cancer Res (2022) 28(16):3473–9. doi: 10.1158/1078-0432.CCR-22-0871 PMC9662895 35675031

[30] Mo H Huang J Xu J Chen X Wu D Qu D. Safety, anti-tumour activity, and pharmacokinetics of fixed-dose SHR-1210, an anti-PD-1 antibody in advanced solid tumours: a dose-escalation, phase 1 study. Br J Cancer (2018) 119(5):538–45. doi: 10.1038/s41416-018-0100-3 PMC6162236 29755117

[31] Xu J Zhang Y Jia R Yue C Chang L Liu R. Anti-PD-1 antibody SHR-1210 combined with apatinib for advanced hepatocellular carcinoma, gastric, or esophagogastric junction cancer: an open-label, dose escalation and expansion study. Clin Cancer Res (2019) 25(2):515–23. doi: 10.1158/1078-0432.CCR-18-2484 30348638

[32] Tran MM Mazzola A Perdigao F Charlotte F Rousseau G Conti F. Primary hepatic angiosarcoma and liver transplantation: radiological, surgical, histological findings and clinical outcome. Clin Res Hepatol Gastroenterol (2018) 42(1):17–23. doi: 10.1016/j.clinre.2017.02.006 28416360

[33] Zhou N Jiang M Li T Zhu J Liu K Hou H. Anlotinib combined with anti-PD-1 antibody, camrelizumab for advanced NSCLCs after multiple lines treatment: an open-label, dose escalation and expansion study. Lung Cancer (2021) 160(undefined):111–7. doi: 10.1016/j.lungcan.2021.08.006 34482102

